# Epidemiology of Rare Hereditary Diseases in the European Part of Russia: Point and Cumulative Prevalence

**DOI:** 10.3389/fgene.2021.678957

**Published:** 2021-08-30

**Authors:** Rena A. Zinchenko, Eugeny K. Ginter, Andrey V. Marakhonov, Nika V. Petrova, Vitaly V. Kadyshev, Tatyana P. Vasilyeva, Oksana U. Alexandrova, Alexander V. Polyakov, Sergey I. Kutsev

**Affiliations:** ^1^Research Centre for Medical Genetics, Moscow, Russia; ^2^Department of Public Health Research, N.A. Semashko National Research Institute of Public Health, Moscow, Russia

**Keywords:** genetic epidemiology, rare hereditary diseases, point prevalence, cumulative prevalence, Russia

## Abstract

The issue of point prevalence, cumulative prevalence (CP), and burden of rare hereditary diseases (RHD), comprising 72–80% of the group of rare diseases, is discussed in many reports and is an urgent problem, which is associated with the rapid progress of genetic technology, the identification of thousands of genes, and the resulting problems in society. This work provides an epidemiological analysis of the groups of the most common RHDs (autosomal dominant, autosomal recessive, and X-linked) and their point prevalence (PP) and describes the structure of RHD diversity by medical areas in 14 spatially remote populations of the European part of Russia. The total size of the examined population is about 4 million. A total of 554 clinical forms of RHDs in 10,265 patients were diagnosed. The CP for all RHDs per sample examined was 277.21/100,000 (1:361 people). It is worth noting that now is the time for characterizing the accumulated data on the point prevalence of RHDs, which will help to systematize our knowledge and allow us to develop a strategy of care for patients with RHDs. However, it is necessary to address the issues of changing current medical classifications and coding systems for nosological forms of RHDs, which have not kept pace with genetic advances.

## Introduction

The problem of rare diseases (RDs) and their number, birth, point, and cumulative prevalence are actively discussed by many researchers, and this is important for public health and society. Criteria for the definition of “rare diseases” differ from country to country depending on legislation. A review by Richter et al. (2015) provides data on 296 definitions of RDs from 1,109 organizations (Richter et al., 2015) and confirms their quantitative differences in different countries. European legislation defines a prevalence threshold of 1 per 2,000 persons. The United States in 1983 defined the threshold for RD as <200,000 affected people in the country (currently 1 in 1,800 people). Japan considers any disease affecting less than 50,000 people in the country as rare, which is equivalent to less than 1 in 2,500 people. In Russia, one patient per 10,000 people in the population is a sufficient measure for a disease being rare (European Union (EU), 2000; Donnart et al., 2013; Richter et al., 2015; Ferreira, 2019; Wakap et al., 2020). RDs are diagnosed in all fields of medicine and occur in all demographic groups (Pariser and Gahl, 2014).

There is a variation and steady increase in the reported RDs according to the main available sources. The Online Mendelian Inheritance in Man (OMIM) database contains 6,806 phenotypes with known genetic nature^1^. According to the Orphanet portal of RDs, about 10,500 RDs are currently registered^2^. A very detailed analysis of the known number, point, and cumulative prevalences of RDs has been performed by several teams (Ferreira, 2019; Wakap et al., 2020). Reviews on RD number analysis and point prevalence estimation cite data from the “Epidemiology section of Orphanet^3^”. It has been shown that 84.5% of the analyzed diseases from the Orphanet database have a point prevalence less than 1 per 1,000,000. However, 77.3–80.7% of the burden of RDs in the population falls on a limited number of diseases, representing only about 4.2% of all identified diseases, with a point prevalence of one to five per 10,000 of the population (Wakap et al., 2020). Ferreira estimates the burden of RDs with manifestation at different periods of life as 6.2% of the total population (Ferreira, 2019). A more conservative estimate by Wakap et al. (2020) demonstrates the cumulative prevalence of RDs in the population to be 3.5–5.9%.

According to various researchers, 72–80% of RDs have a genetic cause, some of which have already been confirmed (Richter et al., 2015; Wakap et al., 2020). The widespread introduction of technologies of whole genome and/or whole-exome analysis into practical healthcare has significantly increased the number of genetically determined diseases in the structure of human morbidity. While by 2012 the molecular nature was identified for 3,650 nosological forms, there has been a greater increase in genetically determined diseases over the past 5 years. According to annual observations of OMIM statistics in 2016, molecular nature was confirmed for 5,888 diseases, in 2017—6,087 (+199), in 2018—6340 (+253), in 2019—6572 (+232), and in 2020—6,800 (+228), i.e., genetic nature is established for an additional 200–250 diseases each year (see text footnote 1). Most of the newly reported forms are rare and found in single families.

Since genetic diseases constitute a high percentage in the RD group, it is advisable to assess the point prevalence of monogenic hereditary diseases in the modern population based on actual data from a specific population survey. This article provides an epidemiological analysis of the point prevalence of rare monogenic hereditary diseases (RHDs) in geographically remote populations of the European part of Russia.

## Results

The population of 96 rural areas, 86 small towns, and urban-type settlements were surveyed during this study. Data on patients with presumptive RHDs were obtained using a questionnaire (Zinchenko et al., 2020b) from medical workers from 125 medical clinics and 2,056 rural ambulant clinics. More than 45,000 patients with various presumably hereditary conditions, including patients with structural chromosomal changes, multiple congenital malformations, and isolated anomalies, were examined in total. A total of 554 clinical forms of RHDs were verified in 10,265 patients (including 4,270 children patients).

Table 1 shows the number of identified patients with RHDs in the regions and the variation of the cumulative prevalence for administrative districts within particular regions.

**TABLE 1:** Number of identified patients with RHDs and variation of cumulative prevalence for administrative districts within particular regions (min/max).

Region of the Russian Federation	Surveyed population (number of districts)	Number of identified patients with RHDs	Variation of cumulative prevalence for districts (min-max)
**Central part of Russia**			
Kostroma region	444,476 (10)	673	1:121-1:545
Kirov region	286,600 (11)	589	1:83-1:548
Bryansk region	88,200 (1)	133	1:324-1:422
Tver region	75,000 (2)	131	1:260-1:405
Republic of Mari El	276,000 (7)	630	1:78-1:286
Chuvash Republic	264,419 (6)	679	1:150-1:550
Republic of Udmurtia	267,655 (6)	794	1:78-1:375
Republic of Tatarstan	264,098 (8)	1516	1:88-1:350
Republic of Bashkortostan	250,110 (8)	1192	1:88-1:389
**Northern part of Russia**			
Arkhangelsk region	40,000 (5)	104	1:150-1:281
**Southern part of Russia and North Caucasus**			
Krasnodar territory	426,600 (6)	740	1:202-1:556
Rostov region	497,460 (12)	1481	1:165-1:340
Republic of Adygea	112,400 (4)	233	1:236-1:387
Republic of Karachay-Cherkessia	410,368 (10)	1857	1:85-1:405
**Average**	**3,703,018 (96)**	10265	

The cumulative prevalence for all RHDs in the sample examined was 277.21 per 100,000 (Zinchenko et al., 2001a,b,^2007^, ^2009^), 558.71 per 100,000 children (Zinchenko et al., 2019, 2020a). The differentiation in the values of cumulative prevalence by region was explained by the peculiarities of the genetic structure of various populations. In the sample under consideration, the main factor of microevolution is genetic drift, migration processes with little influence of natural selection. These results were demonstrated in studies on the role of the genetic structure in the formation of cumulative prevalence in every population (Zinchenko et al., 2000, 2009, 2020b). The number of familial cases averaged 57.82%. There were no statistically significant differences between the cumulative prevalence of AD and AR pathology in the groups of men and women (*p* > 0.05).

**FIGURE 1:**
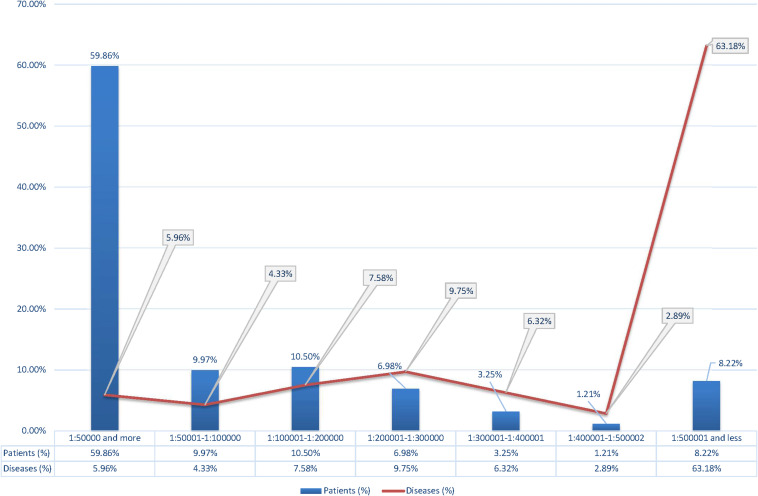
Percentage of patients and diseases according to the point prevalence values.

After that, we have analyzed the point prevalence values and the number of diseases by groups in the surveyed populations (Table 2). The highest number of patients was found in point prevalence class 1 (1:50,000 and more) (59.46%) with the autosomal dominant (AD) type of inheritance—the autosomal recessive (AR) type of inheritance was observed in 34.66% of patients, the X-linked (XL) type of inheritance in 7.88%. The same ratio is observed in the analysis of the number of detected diseases: with the AD type of inheritance—48.01%; with AR—41.34%; and with XL—10.65%. Analysis of the distribution of the number of patients according to the point prevalence values showed that 33 diseases accounted for most of the patients (59.86%), representing only 5.96% of the total number of diseases (Figure 1). The class of RHDs with a point prevalence of “1:500,001 and less” represents 350 diseases (63.18% of all detected RHDs) with 844 patients (8.22% of the patients): 6.85% with the AD type of inheritance, 10.40% with the AR type, and 8.65% with the XL type.

**TABLE 2:** Distribution of patients with AD, AR, and X-linked inheritance patterns of RHDs depending on the point prevalence values and the number of diseases by groups^1^.

Point prevalence	AD		AR		XL		Total	
	Abs. num. of patients (%)	Num. of diseases (%)	Abs. num. of patients (%)	Num. of diseases (%)	Abs. num. of patients (%)	Num. of diseases (%)	Abs. num. of patients (%)	Num. of diseases (%)
1:50,000 and more	3,358 (56.93%)	17 (6.39%)	2,253 (63.32%)	11 (4.80%)	534 (66.01%)	5 (8.47%)	6,145 (59.86%)	33 (5.96%)
1:50,001–1:100,000	742 (12.58%)	16 (6.02%)	205 (5.76%)	5 (2.18%)	76 (9.39%)	3 (5.08%)	1023 (9.97%)	24 (4.33%)
1:100,001–1:200,000	643 (10.90%)	26 (9.77%)	363 (10.20%)	10 (4.37%)	72 (8.90%)	6 (10.17%)	1,078 (10.50%)	42 (7.58%)
1:200,001–1:300,000	481 (8.16%)	34 (12.78%)	193 (5.42%)	14 (6.11%)	43 (5.32%)	6 (10.17%)	717 (6.98%)	54 (9.75%)
1:300,001–1:400,001	214 (3.63%)	22 (8.27%)	110 (3.09%)	11 (4.80%)	10 (1.24%)	2 (3.39%)	334 (3.25%)	35 (6.32%)
1:400,001–1:500,002	56 (0.95%)	7 (2.63%)	64 (1.80%)	8 (3.49%)	4 (0.49%)	1 (1.69%)	124 (1.21%)	16 (2.89%)
1:500,001 and less	404 (6.85%)	144 (54.14%)	370 (10.40%)	170 (74.24%)	70 (8.65%)	36 (61.02%)	844 (8.22%)	350 (63.18%)
Total	5,898	266	3,558	229	809	59	10,265	554

*^1^OMIM—Online Mendelian Inheritance in Man; PS—Phenotypic Series for OMIM with heterogeneity of the disease; AD—autosomal dominant type of inheritance; AR—autosomal recessive type of inheritance; XL—X-linked type of inheritance. Point prevalence for X-linked pathology is for 100,000 of the male population.*

Next, we have analyzed reported diseases and the number of patients with a point prevalence of 1:100,000 or more (Table 3). Table 3 also presents disease prevalence values (adjusted for gender differences) and point prevalence in the child population. It is worth noting that for congenital diseases and those with manifestation in childhood, the prevalence in the child population is much higher than in the general population. Most of the diseases are from heterogeneous groups but having a similar clinical picture. For example, about 100 genetic forms with similar clinical manifestations of the disease are known for retinitis pigmentosa. In our studies, we also determined a wide heterogeneity for this disease based on NGS studies—we diagnosed 35 clinical genetic variants, and they are combined under the clinical diagnosis “retinitis pigmentosa” in Table 3. Some forms were listed separately, i.e., Stargardt disease, because it has characteristic features and is well diagnosed clinically. The same pattern is observed for Charcot–Marie–Tooth disease, the verification of specific nosological forms in most cases being determined by a molecular genetic study. RHDs vary in prevalence, locus, and allelic heterogeneity depending on the geographic region, which is associated with the specific genetic structure of the population. More than 20 rare genetic variants for Charcot–Marie–Tooth disease associated with different loci and represented by single families have been identified in different regions of Russia (Schagina et al., 2007; Khidiyatova et al., 2013; Dadali et al., 2016; Shchagina et al., 2018, 2020; Murtazina et al., 2020).

**TABLE 3:** Reported diseases with high point prevalence of 1:100,000 or more and the number of patients (ranging in decreasing number of total point prevalence).

OMIM #	Diagnosis	Number of patients	Point prevalence per 100,000 (including men/women)	Point prevalence per 100,000 children (newborn-17 years old/boys*)
**Autosomal dominant RHDs**				

**Point prevalence 1:50,000 and more**				

#146700	Ichthyosis vulgaris	646	17.45 (8.61; 8.83)	34.15
PS130000	Ehlers–Danlos syndrome	459	12.40 (6.13; 6.27)	28.00
#148700	Keratosis palmoplantaris	304	8.21 (3.81; 4.40)	14.52
PS118220	Charcot–Marie–Tooth disease	232	6.27 (3.02; 3.24)	5.23
PS116200	Congenital hereditary cataract	215	5.81 (2.89; 2.92)	15.05
#162200	Neurofibromatosis, type I	199	5.37 (2.67; 2.70)	10.60
PS156200	Undifferentiated intellectual disability	177	4.78 (2.40; 2.38)	9.42
#146000	Hypochondroplasia	164	4.43 (2.08; 2.35)	3.14
PS124900	Deafness, autosomal dominant	133	3.59 (1.50; 2.09)	3.14
178300	Ptosis, hereditary congenital	126	3.40 (1.76; 1.65)	12.04
PS268000	Retinitis pigmentosa	122	3.29 (1.62; 1.67)	4.97
PS174200	Polydactyly, postaxial, type A1	112	3.02 (1.57; 1.46)	8.50
151900	Lipomatosis, multiple	108	2.92 (1.38; 1.54)	0
#154700	Marfan syndrome	105	2.84 (1.40; 1.48)	8.23
PS166200	Osteogenesis imperfecta	100	2.70 (1.32; 1.38)	7.98
#185900	Syndactyly, type I	84	2.27 (1.08; 1.19)	7.46
PS163950	Noonan syndrome 1	72	2.03 (0.85; 1.12)	5.40

**Point prevalence 1:500,01–1:100,000**				

#160900	Dystrophia myotonica 1	65	1.76 (0.80; 0.95)	1.05
181800	Scoliosis, idiopathic	69	1.86 (0.86; 1.00)	5.10
#133700	Exostoses, multiple, type I	56	1.51 (0.81; 0.70)	4.19
#100800	Achondroplasia	54	1.46 (0.70; 076)	4.19
#143100	Huntington disease	51	1.38 (0.65; 0.73)	0
#120200	Coloboma, ocular	49	1.32 (0.48; 0.85)	4.58
PS310700	Nystagmus, congenital	49	1.32 (0.53; 0.80)	4.58
PS183600	Split-hand/foot malformation 1	45	1.22 (0.23; 0.22)	3.79
PS174400	Polydactyly, preaxial I	44	1.19 (0.57; 0.62)	3.27
PS303350	Spastic paraplegia, autosomal dominant	42	1.13 (0.59; 0.54)	1.57
#110100	Blepharophimosis, ptosis	41	1.11 (0.57; 0.54)	1.83
126070	Albinoidism, oculocutaneous, autosomal dominant	39	1.05 (0.51; 0.54)	0.92
#158900	Facioscapulohumeral muscular dystrophy 1A	39	1.05 (0.49; 0.57)	0.79
PS165500	Optic atrophy 1	38	1.03 (0.57; 0.46)	1.44
#186000	Synpolydactyly 1	37	1.00 (0.46; 0.54)	2.75
#106210	Aniridia	37	1.00 (0.49; 0.51)	3.27

**Autosomal recessive RHDs**				

**Point prevalence 1:50,000 and more**				

PS220290	Deafness, autosomal recessive	776	20,96 (9.91; 11.05)	59.27
PS249500	Undifferentiated intellectual disability	431	11.64 (5.40; 6.24)	29.31
PS251200	Microcephaly, primary autosomal recessive	155	4.19 (2.11; 2.08)	17.14
PS268000	Retinitis pigmentosa	150	4.05 (1.92; 2.13)	2.88
#261600	Phenylketonuria	145	3.92 (2.00; 1.92)	15.05
PS116200	Congenital hereditary cataract	105	2.84 (1.40; 1.43)	8.24
#242100	Ichthyosiform erythroderma, congenital	84	2.27 (1.08; 1.19)	5.23
PS253600	Muscular dystrophy, limb-girdle	83	2.24 (1.11; 1.13)	1.70
PS203100	Albinism, oculocutaneous	81	2.19 (1.05; 1.13)	7.85
#253300	Spinal muscular atrophy, types I–III	72	2.03 (1.01; 1.01)	8.23
PS262400	Growth hormone deficiency	72	2.03 (1.02; 1.00)	1.70

**Point prevalence 1:50,001–1:100,000**				

PS276900	Usher syndrome	52	1.40 (0.68; 0.73)	2.36
PS204000	Leber congenital amaurosis	44	1.19 (0.50; 0.66)	1.44
#248200	Stargardt disease 1	43	1.16 (0.62; 0.57)	1.83
#604379	Hypotrichosis, total, Mari type	39	1.05 (0.52; 0.53)	1.75
#219700	Cystic fibrosis	37	1.00 (0.49; 0.51)	4.45

**X-linked RHDs**				

**Point prevalence 1:50,000 and more**				

PS309530	Undifferentiated intellectual disability, X-linked	226	12.21 (12.21; 0)	30.09*
#308100	Ichthyosis, X-linked	124	6.70 (6.70; 0)	15.70*
#306700	Hemophilia A	78	4.21 (4.21; 0)	12.04*
#310200	Muscular dystrophy, Duchenne type	55	2.97 (2.97; 0)	10.21*
PS310700	Nystagmus, congenital, X-linked	51	2.75 (2.75; 0)	11.51*

**Point prevalence 1:50,001–1:100,000**				

#305400	Faciogenital dysplasia	32	1.73 (1.67; 0)	7.59*
#300376	Muscular dystrophy, Becker type	24	1.30 (1.30; 0)	1.24*
#302800	Charcot–Marie–Tooth disease, X-linked dominant	20	1.08 (0.53; 0.55)	4.71

*Point prevalence for X-linked pathology is estimated for 100,000 boys.

Differential diagnosis of “undifferentiated intellectual disability” is particularly difficult, and work is being done in this direction identifying new genes and genetic variants, most of which we identify in single families (Levchenko et al., 2019). In our sample, the majority of cases were familial.

In Russia, we have identified allelic heterogeneity, and regional and ethnic peculiarities, for most diseases included in newborn screening (Petrova et al., 2016, 2019a,2019b; Gundorova et al., 2018). Our studies identified the highest prevalence of phenylketonuria around the world (birth prevalence 1:850 newborns) in one region of the Russian Federation, and it is associated with the spread of a specific mutation (p.R261^∗^ in the *PAH* gene) associated with the founder effect (Gundorova et al., 2018). The study of allelic heterogeneity is necessary because the varying significance of different genetic variants leads to different clinical courses of diseases and treatment correction (Petrova et al., 2016, 2019a,2019b; Gundorova et al., 2018). For some diseases, the analysis of genotype–phenotype correlations and the mutation spectrum has identified peculiarities of the clinical course of the disease (Andreeva et al., 2016; Marakhonov et al., 2019; Petrova et al., 2019b; Vasilyeva et al., 2021).

In addition to RHDs that are frequent for all populations of the Russian Federation and Europe, regionally specific diseases have been identified. Our study identified diseases endemic to specific regions of the Russian Federation—several types of hypotrichosis (Kazantseva et al., 2006; Zernov et al., 2016), osteopetrosis (Bliznetz et al., 2009), and a number of RDs identified in the Russian Federation and worldwide in single cases—primary microcephaly (Marakhonov et al., 2018), gnathodiaphyseal dysplasia (Andreeva et al., 2016), metatropic dysplasia (Timkovskaya et al., 2016), etc.

The assessment of the cumulative prevalence and burden of RHDs according to the common medical classification of diseases is important for public health and public policy in Russia and in European countries.

We analyzed the diversity and point prevalence of RHDs according to the conventional classification of the disease by types of affected organs and systems according to their main clinical manifestations—neurological syndrome, ophthalmic syndrome, genodermatoses, skeletal syndrome, hereditary syndrome, and other pathology (hereditary diseases of metabolism, blood, hearing, etc.). Table 3 presents the number of patients, the number of diseases, and the point prevalence of RHDs distributed according to the medical classification of diseases.

Analysis of Table 4 shows that the maximum number of patients (23.56%) was found to have neurological and psychiatric pathology, the cumulative point prevalence of which is 65.30/100,000 or 1:1530 people. The next most numerous groups were hereditary syndromes—18.04% (cumulative point prevalence 50.01/100,000 or 1:2,000) and other pathology (hereditary diseases of metabolism, blood, hearing, etc.)—17.11% (47.42/100,000 or 1:2,109 people). For the first two groups of diseases, the maximum incidence was 18.38 and 36.94%, respectively.

**TABLE 4:** Structure of the diversity and point prevalence of the RHDs in accordance with the main medical classification of diseases.

Types of the hereditary disease	Patient data		Disease data	
	Abs. num. of patients (%)	Point prevalence per 100,000	Num. of diseases	%
Neurological and psychiatric	2418 (23.56%)	65.30	102	18.38%
Ophthalmic	1524 (14.85%)	41.16	73	13.15%
Genodermatoses	1510 (14.71%)	40.78	39	7.03%
Skeletal	1205 (11.74%)	32.54	87	15.68%
Hereditary syndromes	1852 (18.04%)	50.01	204	36.94%
Other pathology (hereditary diseases of metabolism, blood, hearing, etc.)	1756 (17.11%)	47.42	49	8.83%

Analysis of the age category of patients with RHDs showed that the majority of patients (about 45.3%) belonged to the category of childhood (from newborn to 17 full years), despite the fact that the share of the child population in the regions is only 20.64%; for the reproductive and post-reproductive age, 54.7% of all patients. The results obtained demonstrate the need to develop preventive programs specifically among children.

## Discussion

The issue of point prevalence, cumulative prevalence, and burden of RHDs is discussed in many articles and is an urgent problem that is associated with the rapid progress of genetic technology, the identification of thousands of genes, and the problems in society, public health, and social structures of states. According to OMIM statistics, genetic nature has been determined for 6,806 phenotypes so far, and the genetic nature is established for another 200–250 diseases each year (see text footnote 1). Current medical classifications have not kept pace with genetic progress; nosological forms of RHDs are underrepresented in coding systems (e.g., International Classification of Disease, ICD-11). Data collection from different researchers lacks a unified methodological approach, which makes comparative analysis difficult. Insufficient organization of the process, lack of knowledge, lack of diagnostic expertise, lack of information on point prevalence, distribution of RHDs by medical areas, and cumulative burden of RHDs prevent the full development of a public health strategy. In addition to public health questions, it is a priority to provide medical care for patients with specific RHDs and to identify the most common RHDs for the necessary prioritization and development of regional, national, and global health programs.

This study presents an epidemiological analysis of the point prevalence of RHDs based on actual material based on a total survey of several regions of the European part of Russia. Russia is a multinational country with a population of 146,238,185, which makes it difficult to choose a unified strategy in public health. Fourteen regions (Northern, Central, and Southern Russia) were chosen for the survey and subsequent analysis, both populations with different ethnic extractions, and regions of a single ethnic origin. Selection of different ethnically diverse territories in a multinational country was necessary to be able to identify groups of the most common RHDs, determine point prevalence, and describe the structure of RHDs diversity by medical areas. The study was performed by a single team, and the collection and processing of material remained unchanged throughout the study.

Despite the listed limitations of the method (see section “Features and Limitations of the Method”) in our study, every 351 people have a hereditary disease. Remarkably higher values of cumulative prevalence were obtained in children—the proportion of children out of the total number of patients with RHDs was 43.3%. This number is remarkably higher than the proportion of children in the general population (20.64%). The main reason for this age distribution is that up to 70% of RHDs manifest in childhood according to the Orphanet database. However, the distribution according to the inheritance type was uneven: 39.17% with AD pathology, 45.24% with AR and 52.28% X-linked. Moreover, the proportion of child patients (of pre-productive age) varied from 38 to 51% in different surveyed populations (Zinchenko et al., 2019, 2020a). The proportion of patients in the reproductive age (18–45 years) out of the total number of patients with RHDs was 37.2% (from 31.91 to 40% by population); for the post-reproductive age (46 and older), only 17.5% (variation 17–22%) out of all patients (Elchinova et al., 2017). Analysis of the sample showed that some diseases do not occur in older age groups because they have high mortality in childhood and middle age (the rate is not constant and varies depending on the population, the causing gene, and the mutation). The lower point prevalence in the older age group is mostly due to a milder and stable course of a limited number of RHDs, with fitting approaching 1, which reduces their relevance and referral to medical facilities.

The number of familial cases and individual cases in family (including sporadic cases for AD pathology) varied depending on the type of inheritance and the average size of the family in a particular region. Among families with the AD type of inheritance, the familial case rate ranged from 70 to 80% in different populations. In families with the AR type, familial cases were 26–34%; for those X-linked, 15–20%. The distribution of men and women did not differ for autosomal pathology. We assume that due to the limitations detailed in the materials and methods, the data obtained may be an underestimate. However, it should be noted that there was a variation by region and by location. The cumulative prevalence and diversity of RHDs was determined for each region, and the most common RHDs were identified. A general disease registry was then compiled. The analysis showed that a single class of the most common RHDs was identified for all populations, with insignificant variation by region for most diseases. The most significant differences in prevalence were found among the child population for congenital and hereditary diseases with early onset. The largest number of patients was detected in the point prevalence class “1:50,000 and more”—33 clinical forms of RHDs (5.96% of the total number of detected diseases) accounting for 59.86% of the patients. Most of the diseases are from heterogeneous groups of RHDs, and there is locus and allelic heterogeneity by region. The smallest number of patients, 8.22%, was identified in the class of RHDs with a point prevalence of “1:500,001 or less”—350 diseases (63.18% of all identified RHDs). These RHDs were mostly detected in single families.

Similar data were obtained by Wakap et al. (2020) when analyzing the Orphanet database epidemiological data (see text footnote 3) for RDs, not all of which are of monogenic nature, but only some of them (70–80%) (Wakap et al., 2020). Their study showed that out of 5,304 diseases—84.5% have a point prevalence <1/1,000,000, and 77.3–80.7% of the burden of RDs in the population accounts for 4.2% (*n* = 149) of the diseases. The analysis performed in our study on a real contemporary population demonstrates the rarity of most RHDs affecting single families and highlights the difficulty of detection and diagnosis by physicians.

Wakap et al. (2020) also highlighted that it would be a benefit to perform an analysis of distribution of diseases in real populations according to the medical classification for organization of real medical care. An analysis of the pattern of diversity of RHDs by medical specialties, which has been performed in the current study, showed a predominance of patients (41.60% of patients) with neurological, psychiatric, and hereditary syndromes with a point prevalence of 115/100,000 people. Most diseases of neurological, psychiatric, and hereditary syndromes; hereditary metabolism diseases; and hereditary blood diseases are characterized by reduced fitness, multisystemic lesions, disability, and reduced life expectancy and quality because of the lack of effective treatment. Most hereditary skin, eye, ear, skeletal, and treatable metabolic diseases could affect quality of life (including possible disability) but have adaptation in society and average life expectancy. The findings require a comprehensive public health approach.

## Conclusion

In conclusion, the present time is characterized by the accumulation of data on the point prevalence of RHDs, and this will help to systematize our knowledge and allow us to develop a strategy of healthcare for patients with RHDs.

## Materials and Methods

We analyzed long-term studies (1985–2020) on the epidemiology of rare monogenic hereditary diseases in the European part of Russia. This approach allows us to identify disease peculiarities in a given region and provides insight into the diversity of rare hereditary pathologies and their point prevalence.

### Surveyed Population

**FIGURE 2:**
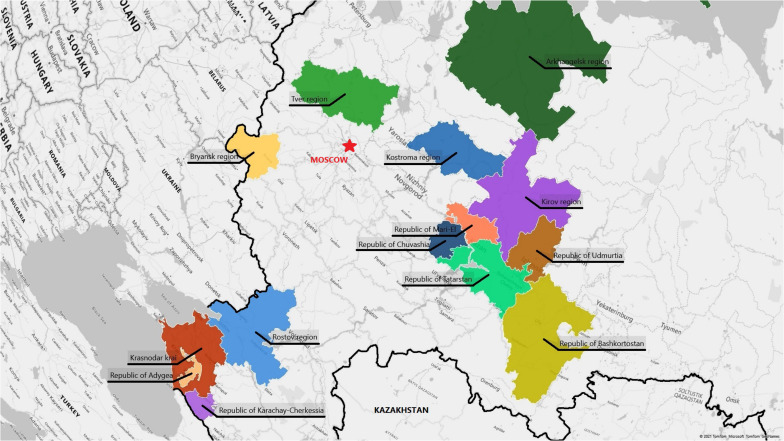
Map of Russian Federation with regions included into the study. The border of the Russia is in bold.

Russia is a multinational state with more than 190 ethnic groups, with Russians constituting about 80%. The study covered various remote areas of European Russia—Central, Northern, Southern, and the North Caucasus. The relative location of the studied regions is shown in Figure 2. The selection of survey areas in each region was focused on the indigenous population (the history and migration flows of population formation were studied). Table 5 shows the populations surveyed, the size of child populations, the ethnic composition of the populations, number of medical organizations (hospital, medical ambulance, paramedic, and obstetric centers), and the number medical workers (physicians, nurses, paramedics) participated in the study. The total size of the surveyed population was 3,703,018 people from 96 rural districts, including 764,260 children (from newborns to 17 full years of age). The sex distribution of the total surveyed population was 1,716,298 (46.35%) men/1,987,087 (53.66%) women^4^. There was an uneven distribution in age: up to 35 years 1:1 (M/W), 36–50 years 0.8:1 (M/W), and the post-reproductive period 0.6:1 (M/W). In total, from the surveyed regions, the study involved 2,181 medical organization and 6,370 medical workers. Each of the studied districts of the particular region has one central district hospital, which includes in-patient and medical ambulance, and several rural ambulances and paramedic–obstetric centers in the villages. The number of rural medical organizations depends on the number of villages in the region and is determined by the Department of Medical Statistics of the hospital. The medical workers permanently residing in the area has full medical information about the attached population.

**TABLE 5:** Number, ethnic composition of the surveyed populations, number of organization, and medical workers who participated in the study.

Region of the Russian Federation	Size of the region population	Surveyed population/size of children (number of districts)	Main ethnic groups	Number of medical organization which participated in the study	Number medical workers who participated in the study
**Central part of Russia**					
Kostroma region	637,267	444,476/80,895 (10)	Russians (>90%), other	177	586
Kirov region	1,272,109	286,600/51,051 (11)	Russians (>90%), other	201	619
Bryansk region	1,200,187	88,200/14,906 (1)	Russians (>90%), other	44	64
Tver region	1,269,636	75,000/51051 (2)	Russians (>90%), other	38	81
Republic of Mari El	728,000	276,000/51051 (7)	Maris (62.16%), Russians (32.14%), other (5.7%)	145	271
Chuvash Republic	1,314,000	264,419/67,863 (6)	Chuvashes (67.59%), Russians (25.27%), other (7.14%)	241	504
Republic of Udmurtia	1,570,000	267,655/60,197 (6)	Udmurts (58%), Russians (31.43%), other (10.57%)	272	513
Republic of Tatarstan	3,838,230	264,098/57,648 (8)	Tatars (79.24%), Russians (10.24%), other (10.52%)	253	577
Republic of Bashkortostan	4,093,795	250,110/64,935 (8)	Bashkirs (69.48%), Russians (14.14%), other (16.38%)	255	675
**Northern part of Russia**					
Arkhangelsk region	1,128,099	40,000/7,440 (5)	Russians (>90%), other	22	172
**Southern part of Russia and North Caucasus**					
Krasnodar territory	5,124,400	426,600/78,921 (6)	Russians (>90%), other	153	446
Rostov region	4,406,700	497,460/101,845 (12)	Russians (>90%), other	161	582
Republic of Adygea	447,000	112,400/21,581 (4)	Adygeans (57.83%), Russians (36.83%), other (5,34%)	56	124
Republic of Karachay-Cherkessia	470,000	410,368/90,739 (10)	Karachays (39.58%), Russians (32.84%), Cherkess (12.38%), Abazins (8.11%), Nogais (3.59%), other (3.5%)	163	1156
**Average**	**31,273,391 (21.39%)**	**3,703,018/764,260 (96)**		**2 181**	6370

### Survey and Protocol

The survey was carried out by a single team of the Research Centre for Medical Genetics (RCMG) in accordance with the protocol of medical and genetic examination of small populations described previously in detail (Zinchenko et al., 2020b). A survey of the investigated populations was conducted regardless of ethnicity, age, and gender structure. The protocol includes the study of populations through different genetic systems simultaneously: (1) population survey, study of the point and cumulative prevalence of hereditary diseases in the particular population; (2) study of the genetic structure using standard methods of population statistics (analysis of the marriages and migration structure, demographic data, analysis of the frequency of surnames to obtain indicators of random inbreeding through isonymy); and (3) study of the genetic structure through the neutral DNA loci of the nuclear genome.

**FIGURE 3:**
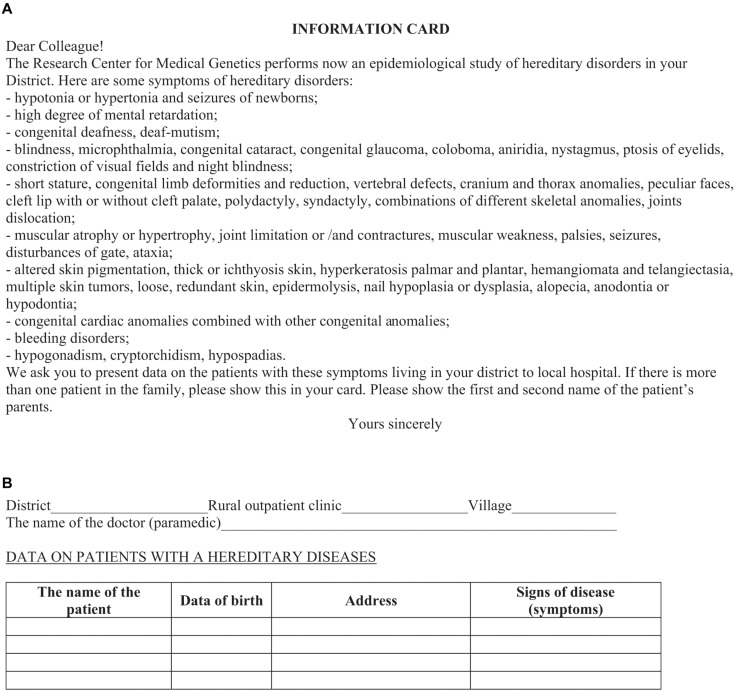
Information card. Front **(A)** and reverse **(B)** sides.

The survey was conducted in three consecutive steps in accordance with the protocol. At the first step, a lecture course is conducted for all medical workers of each surveyed districts of regions (physicians of different specialties, nurses, paramedics) to explain the goals and objectives of the study. During the lecture, the medical personnel are given an information card–questionnaire (Figure 3A) with request for filling in the information on patients (Figure 3B), including those at the initial stage of disease and with minimal clinical manifestations^5^. The questionnaire contains easily detectable clinical symptoms of RHDs, almost each of which is characteristic for a group of diseases (isolated, syndromic forms). This protocol allowed the detection of the maximum possible number of nosological forms of RHDs known to date. The card–questionnaire with the listed symptoms allows to reveal practically all “portrait” syndromes. Registration of families presumably with RHDs was done through affected persons in the family. The “Multiple registration” method was used (Cavalli-Sforza and Bodmer, 1999). In addition to the questionnaire card, other sources of information are involved: (i) data on disabled persons (from childhood to adults) in the district provided by the hospital; (ii) information from special schools for the blind and visually impaired, deaf, and hard of hearing and schools for children with intellectual disability; and (iii) data from the genetic counseling unit. Thus, registration of the same patient was possible from several sources of information, i.e., being multiple, but recorded in a single database as one case for further examination. A one-time examination of congenital and hereditary pathology in each region is carried out. In the aggregate, the detection rate of patients from all sources of registration reaches 80%.

At the second step, the patients were examined by clinical geneticists from the RCMG. The patient with the presumed RHDs is given a medical card, which contains personal information of the proband and his family members, a brief medical history, genealogical data, and detailed phenotyping data. In the process of data collection, pedigrees and the possibility of consanguinity were analyzed. Consanguinity was revealed in rare cases. If necessary, a cytogenetic study is conducted to rule out chromosomal abnormalities. In complicated cases, additional examinations were prescribed for patients to be able to verify the diagnoses (biochemical, radiological, electromyographic, and other methods). As a result of this step, a significant proportion of patients (usually, more than half) from different sources is excluded from the sample because of the external cause of the disease (injuries, infections, isolated congenital pathology, etc.). The remainder families represent a list of families presumably with RHDs for further research.

At the third step, clinical investigations were performed by specialists from leading federal research institutes (geneticist, neurologist, ophthalmologist, dermatologist, pediatrician, otolaryngologist, and orthopedist), which ensured unification of diagnostic criteria. In some cases, blood is collected from patients for molecular genetic diagnosis. Written informed consent was obtained from all identified and examined families for voluntary participation in the study. From 1 to 5% of patients refuse to be examined for various reasons. The study was approved by the Ethical Committee of the Research Centre for Medical Genetics (Protocol No. 17/2006 dated 02.02.2006).

### Statistical Methods

Given the heterogeneity of many diseases, the type of inheritance was also confirmed by segregation analysis used in multiple-family registration (Cavalli-Sforza and Bodmer, 1999). Segregation frequency is calculated by Weinberg’s proband method (Morton, 1959). Using this method, segregation frequencies are calculated (separately for families with AD and AR pathology) by the ratio of probands; for actually detected patients in families after examination, probability of registration. In our case, the calculated segregation frequencies corresponded to the expected ones—0.25 for the AR type of inheritance and 0.5 for AD. However, it should be noted that the probability of registration differed from 100% and was 83% for the group of families with AR pathology and 72% for AD. The results suggest that irrespective of the total screening performed, a certain number of patients might not have been registered by us.

Point prevalence was calculated according to the procedural document on epidemiology of RDs in Orphanet (2019) as the number of reported cases in the population at a given time point per 100,000 people (all age categories were considered) (European Union (EU), 2009). The prevalence of X-linked pathology was calculated for the male population in the surveyed regions. The average male population in the regions was 46.44% (variation 45.6–46.9%).

A nosological registry of detected RHDs based on clinical diagnoses was compiled. For RHD diversity analyses, seven groups were selected with a point prevalence interval of (i) 1:50,000 and more frequently, (ii) 1:50,001–1:100,000, (iii) 1:100,001–1:200,000, (iv) 1:200,001–1:300,000, (v) 1:300,001–1:400,001, (vi) 1:400,001–1:500,002, and (vii) 1:500,001 and less frequently.

Based on clinical manifestations, we additionally analyzed point prevalence according to the generally accepted classification of diseases: neurological syndrome, ophthalmic syndrome, genodermatoses, skeletal syndrome, hereditary syndrome, and other pathology (hereditary diseases of metabolism, blood, hearing, etc.).

The methods for collection and processing of medical genetic material remained unchanged throughout all the studies, which allows comparison of newly obtained data with results from the previously surveyed populations of the country.

### Molecular Genetic Analysis

Confirmatory DNA diagnostics was carried out in the laboratories of the RCMG: Laboratory of Genetic Epidemiology (head—R.A. Zinchenko), Laboratory of Epigenetics (head—Sci. V.V. Strelnikov), and Laboratory of DNA Diagnostics (head—A.V. Polyakov). A variety of methods were used for DNA diagnosis—Sanger sequencing, MLPA, RFLP, AFLP, and whole-exome sequencing—depending on the studied nosology according to the protocols published elsewhere by the authors of the current manuscript (Zinchenko et al., 2020b).

### Features and Limitations of the Method

Monogenic hereditary diseases listed in the OMIM and Orphanet databases with AD, AR, and X-linked (XL) types of inheritance were included in the analysis. Patients with mitochondrial disorders and chromosomal rearrangements were excluded from the analysis after cytogenetic and molecular genetic studies.

Some patients may not be identified or missed because of the following reasons: (i) patients with subclinical forms of the disease; (ii) patients in the initial stage of the disease with late onset; (iii) patients who refused from examination (from 1 to 5%) for various reasons: observation and treatment by a specific physician, unwillingness to disclose their diagnosis, and others; (iv) patients who are not registered in a medical organization of the region; (v) patients who have not passed this examination and do not live in the region (even in familial cases of the disease); (vi) patients who died by the time of the survey; (vii) child patients under 1 year of age with severe hereditary metabolic diseases unable to pass the survey; and (viii) we also assume that due to the increase in the number of confirmed phenotypes at the genetic level in recent years, the recognition of these diseases by physicians of various specialties is not yet possible. There is a lack of knowledge and diagnostic experience, which must inevitably lead to the omission of sporadic cases of rare hereditary diseases. Doctors in all countries face these problems.

The resulting values of the probability of registration are 83% for the group of families with AR pathology and 72% for AD. We were also unable to estimate the annual incidence (number of newly diagnosed cases in a population within 1 year) due to the lack of this information in medical organizations. In consequence of the above, certain corrections should be made taking into account the limited number of nosological forms envisaged by our study.

## Data Availability Statement

The data analyzed in this study is subject to the following licenses/restrictions: Data are available upon request. Requests to access these datasets should be directed to RZ, renazinchenko@mail.ru.

## Ethics Statement

The studies involving human participants were reviewed and approved by The Ethical Committee of the Research Centre for Medical Genetics. Written informed consent to participate in this study was provided by the participants’ legal guardian/next of kin.

## Author Contributions

RZ and EG: conceptualization, methodology, visualization, and funding acquisition. RZ, TV, and OA: validation. RZ, NP, VK, AP, and AM: formal analysis. RZ: resources, data curation, and writing—original draft preparation. AM: writing—review and editing. EG and SK: supervision. RZ, EG, and SK: project administration. All authors have read and agreed to the published version of the manuscript.

## Conflict of Interest

The authors declare that the research was conducted in the absence of any commercial or financial relationships that could be construed as a potential conflict of interest. The reviewer MM declared a past co-authorship with the authors RZ, EG, AM, NP, TV, and SK to the handling editor.

## Publisher’s Note

All claims expressed in this article are solely those of the authors and do not necessarily represent those of their affiliated organizations, or those of the publisher, the editors and the reviewers. Any product that may be evaluated in this article, or claim that may be made by its manufacturer, is not guaranteed or endorsed by the publisher.

## Footnotes

1
https://omim.org/statistics/geneMap

2
http://www.orphadata.org/

3
http://www.orphadata.org/cgi-bin/epidemio.html

4
https://infotables.ru/statistika/31-rossijskaya-federatsiya/200-sootnoshenie-chislennosti-muzhchin-i-zhenshchin-rossii-v-2010-i-2002-godakh-tablitsa

5 Transfer of patients’ data was performed in accordance with Article 13 of Federal Law No. 323-FL “On the basics of health protection of citizens of the Russian Federation” which allowed the submission of information about patients in the following cases: the exchange of information by medical organizations, including those deposited in medical information systems, for the purpose of providing medical care, taking into account the requirements of the legislation of the Russian Federation (paragraph 8), as well as for the purpose of accounting and control within the mandatory medical insurance system (paragraph 9) [http://docs.cntd.ru/document/902312609].
