# Corrigendum: Epidemiology of rare hereditary diseases in the European part of Russia: Point and cumulative prevalence

**DOI:** 10.3389/fgene.2022.1019916

**Published:** 2022-09-09

**Authors:** Rena A. Zinchenko, Eugeny K. Ginter, Andrey V. Marakhonov, Nika V. Petrova, Vitaly V. Kadyshev, Tatyana P. Vasilyeva, Oksana U. Alexandrova, Alexander V. Polyakov, Sergey I. Kutsev

**Affiliations:** ^1^ Research Centre for Medical Genetics, Moscow, Russia; ^2^ Department of Public Health Research, N.A. Semashko National Research Institute of Public Health, Moscow, Russia

**Keywords:** genetic epidemiology, rare hereditary diseases, point prevalence, cumulative prevalence, Russia

In the published article, there was an error in the **Funding** statement. The state assignment of the Ministry of Science and Higher Education of the Russian Federation was incorrectly mentioned as a funding source. The correct **Funding** statement appears below:

“This study was supported by the Russian Science Foundation, grant no. 17-15-01051.”

The authors apologize for this error and state that this does not change the scientific conclusions of the article in any way. The original article has been updated.

